# Circular RNA circHMCU promotes breast tumorigenesis through miR-4458/PGK1 regulatory cascade

**DOI:** 10.1186/s41065-023-00275-y

**Published:** 2023-03-23

**Authors:** Shubian Qiu, Lele Zou, Ruimin Qiu, Xin Wang

**Affiliations:** 1 Department of Thyroid and Breast Surgery, Nanyang Second General Hospital, NO. 66, Jianshe East Road, Nanyang, 473000 Henan Province China; 2 Department of Radiotherapy, Nanyang Second General Hospital, Nanyang, 473000 Henan Province China

**Keywords:** Breast cancer, circHMCU, miR-4458, PGK1, Glycolysis

## Abstract

**Background:**

Circular RNAs (circRNAs) are abnormally expressed in breast cancer (BC). However, the biological function and mechanism of circHMCU still need to be further explored.

**Methods:**

The expression levels of circHMCU, miR-4458 and phosphoglycerate kinase 1 (PGK1) were measured by quantitative real-time polymerase chain reaction (qRT-PCR) or western blot. The glucose uptake, lactate production and ATP level were assayed by related commercial kits. Cell Counting Kit-8 (CCK8), 5’-ethynyl-2’-deoxyuridine (EdU) and flow cytometry assays were used to test cell proliferation and apoptosis, respectively. The migratory and invasive abilities were detected by transwell and wound-healing assays. The relationships among circHMCU, miR-4458 and PGK1 were verified by dual-luciferase reporter assay. The function of circHMCU in tumor growth was evaluated by animal studies.

**Results:**

CircHMCU was upregulated in BC tissues and cell lines, whereas miR-4458 was downregulated. For biological experiments, circHMCU knockdown inhibited cell proliferation, migration, glycolysis, while promoted cell apoptosis. CircHMCU bound miR-4458, and miR-4458 targeted PGK1. MiR-4458 inhibition reversed circHMCM knockdown-mediated effects on BC cell malignant behaviors. MiR-4458 overexpression suppressed cell glycolysis, proliferation, and metastasis and promoted apoptosis in BC cells through PGK1 upregulation. Additionally, circHMCU suppressed tumor growth in vivo.

**Conclusion:**

CircHMCU acted as an oncogenic factor by regulating the miR-4458/PGK1 axis in BC.

**Supplementary Information:**

The online version contains supplementary material available at 10.1186/s41065-023-00275-y.

## Introduction

Breast cancer (BC) is one of the most common gynecological malignant tumors and the leading cause of cancer-related deaths worldwide in women [1, 2]. Screening mammography and early clinical diagnosis are associated with an increased BC survival rate [3]. However, not all BC cases can be detected by mammography. Thus, there is an urgent need to find new diagnostic biomarkers for BC.

Circular RNAs (circRNAs) are formed by exonic circularization or intronic circularization, which is a closed loop structure without 5’ caps and 3’ poly tails [4]. Because of high stability and implicated roles in gene regulation, circRNAs have important biological functions in human cancers, including BC [5, 6]. As an example, circNFATC3 has been demonstrated to play a vital role in tumor cell proliferation, migration and metastasis in BC [7]. CircRNAs have been demonstrated to act as molecular sponges to regulate microRNA (miRNA) expression [8]. For example, circHIPK3 was inversely correlated with miR-326 expression and sponged miR-326 to promote BC progress [9]. Moreover, circRNAs can interact with miRNAs and thus take part in the regulation of target gene expression, indicating their activity of endogenous competitive RNA (ceRNA) [10]. For instance, functional experiments showed that suppression of circRPPH1 inhibited BC cells proliferation, military, glycolysis and tumor growth via modulating miR-296-5p/FOXP4 axis [11]. Circ_0084927 promoted BC progression by modulating miR-142-3p to upregulate ERC1 [12]. CircHMCU (also called hsa_circ_0000247 based on the circRNA ID in the database circBase), generated through back-splicing of HMCU pre-mRNA, has established a promoter role in BC and can facilitate BC cell growth and migration by sponging let-7 family [13]. Based on its crucial role in BC metastasis, we focused on circHMCU in this study. Furthermore, a great amount of internal ceRNA mechanisms of circHMCU in regulating BC remain unclear.

There is ample evidence that miRNAs have regulatory functions in breast tumorigenesis by controlling gene expression [14]. MiR-4458 can hamper the proliferation and metastasis of BC by binding CPSF4 [15]. Also, miR-4458 constrained cell migration and the proliferation by targeting SOCS1 in BC cells [16]. Additionally, phosphoglycerate kinase 1 (PGK1) is upregulated in various types of BC [17, 18], and it is a potential tumor promoting factor in BC [19]. However, the relationships among circHMCU, miR-4458 and PGK1 are still undefined.

Glycolysis is a form of glycometabolism, which takes up glucose produced ATP to tumor cell growth. Glycolysis activities contribute to tumor progress and reflect the associated prognosis [20, 21]. Vitamin D treatment can inhibit glycolutic enzymes activities and block glucose uptake in BC cell lines [22]. The glycolytic genes are aberrant expression in tumor cells due to the activation of tumor promoters or inhibition of tumor suppressors. For example, circ_0008039 supports BC tumorigenesis and glycolysis by regulating the miR-140-3p/SKA2 axis [23]. Kim et al*.* reported that GREM1, the antagonist of bone morphogenetic proteins, enhanced the level of HK2 to promote glucose uptake by regulating ROS-Akt-STAT3 axis [24]. However, the correlation between glycolysis and regulation of gene expression needs to be further explored.

In our study, we aimed to explore the functions and relationships of circHMCU, miR-4458 and PGK1 in BC progression through a series of functional experiments.

## Materials and methods

### Clinical tissues and cell lines

A total of 66 pairs of BC tissue samples and adjacent normal tissue samples were obtained from BC patients at the Nanyang Second General Hospital, and all tissues were stored at -80℃. The clinical characteristics of these patients were shown in Table 1. All patients had not undergone any chemotherapy and radiotherapy before surgery. The experiment here got approval from the Ethics Committee of Nanyang Second General Hospital.

**Table 1:** Clinical characteristics of patients with breast cancer (*n* = 66)

Characteristic	Cases
Age	
< 50	38
≥ 50	28
Histologic grade	
I	3
II	45
III	18
Lymph node metastasis	
Yes	35
No	31
T stage	
T1/T2	52
T3/T4	14
N stage	
N0/N1	39
N2/N3	27

BC cell lines (MDA-MB-231 and MCF-7), human embryonic kidney cell line 293 T and human normal mammary epithelial cell line (MCF10A) were obtained from Procell (Wuhan, China). MDA-MB-231 cells were maintained in Leibovitz's L-15 medium (Procell); MCF-7 cells were maintained in Minimum Essential Medium (MEM) medium (Procell); MCF10A cells were maintained in Dulbecco’s modified Eagle’s medium (DMEM)/F12 medium (Procell); 293 T cells were cultured in DMEM medium (Procell). All mediums were added with 10% fetal bovine serum (FBS; Gibco, Thermo Fisher Scientific, Rockville, MD, USA) and 1% penicillin–streptomycin liquid (Gibco). All cells were cultured at 37℃ at 5% CO_2_.

### Quantitative real-time polymerase chain reaction (qRT-PCR)

Total RNA was isolated from cultured cells and clinical tissues by TRIzol reagent (Invitrogen, Carlsbad, CA, USA). Subsequently, RNA (500 ng-1 μg) was reverse transcribed into cDNA by M-MLV RT Reagent (Promega, Madison, WI, USA) or miScript RT Kit (TaKaRa, Dalian, China). The levels of circHMCU, miR-4458 and PGK1 were measured by SYBR Premix Ex Taq II (TaKaRa) and calculated by the 2^−△△Ct^ method. The housekeeping gene (β-actin or U6) acted as an endogenous control. The sequences of primers were displayed in Table 2.

**Table 2:** Primers sequences used for PCR

Name		Primers for PCR (5’-3’)
hsa_circ_0000247	Forward	CTGTTCACGCAGGGGAAACT
	Reverse	AGCAGCTAAGATGTCACTGGC
PGK1	Forward	CCACTGTGGCTTCTGGCATA
	Reverse	ATGAGAGCTTTGGTTCCCCG
HMCU	Forward	CCACTGGGCCTCTCTAAACA
	Reverse	CTCCCACCCTGATTCCAAGT
miR-4458	Forward	TCGGCAGGAGAGGTAGGTGTGGAAGAA
	Reverse	CTCAACTGGTGTCGTGGAG
β-actin	Forward	CTTCGCGGGCGACGAT
	Reverse	CCACATAGGAATCCTTCTGACC
U6	Forward	CTCGCTTCGGCAGCACA
	Reverse	AACGCTTCACGAATTTGCGT

### RNase R assay and actinomycin D assay

For the RNase R experiment, RNA (10 μg) was collected from BC cells and divided into two parts. For RNase R^+^ group, the RNA was treated with RNase R (3 U/μg) (Sigma-Aldrich, St. Louis, MO, USA) at 37 °C for 15 min. For RNase R^−^ group, the RNA was only treated with digestion buffer. The expression levels of circHMCU and linear HMCU were examined by qRT-PCR.

For the actinomycin D experiment, cells were cultivated into 6-well plates with or without Actinomycin D (2 μg/mL) (Sigma-Aldrich) treatment. Then, the cells were collected at 0 h, 4 h, 8 h, 12 h, 24 h, and the levels of circHMCU and linear HMCU were measured by qRT-PCR.

### Cell transfection

The small interfering RNA (siRNA) to circHMCU (si-circHMCU) and si-NC control mock, miR-4458 mimic, inhibitor and related controls (mimic NC and inhibitor NC) were bought from Geneseed (Guangzhou, China). PGK1 encoding sequence was cloned into pcDNA3.1 vector (Invitrogen) to produce PGK1 expression plasmid, and empty vector acted as the negative control. BC cells were transfected with oligonucleotides or plasmids using lipofectamine 3000 transfection reagent (Invitrogen).

### Glucose consumption, lactate production and ATP level assays

Glucose Uptake Colorimetric Assay kit (Biovision, Milpitas, California, USA) was used to measure glucose consumption in culture supernatants of transfected cells. Lactate production in cultured cells was detected by Lactate Assay Kit II (Biovision). The ATP level in cultured cells was determined with ATP assay kit (Beyotime, Shanghai, China).

### Cell Counting Kit 8 (CCK8) assay

Cell proliferation was assayed by CCK8 (Beyotime). After transfection, BC cells (5000 cells) were maintained into 96-well plates and cultured for 0 h, 24 h, 48 h, 72 h. At above time points, a fresh medium containing 10% CCK8 reagent was added to the 96-well plates. After incubation for 2 h, the absorbance of 450 nm was measured with a microplate reader (Thermo Fisher Scientific).

### 5-Ethynyl-20-deoxyuridine (EdU) incorporation assay

For EdU assay, the Cell-Light EdU DNA Cell Proliferation Kit (RiboBio, Shanghai, China) was used to measure cell proliferation. Briefly, BC cells (1 × 10^4^) were cultivated and incubated with 50 nM EdU for 2 h. Subsequently, cells were added 4% paraformaldehyde and stained with Apollo Dye Solution and DAPI. The positive cells were counted with a microscope (Olympus, Tokyo, Japan).

### Apoptosis assay

The Annexin V-fluorescein isothiocyanate (FITC)/propidium iodide (PI) apoptosis detection kit (Solarbio, Beijing, China) was used to evaluate the apoptosis of BC cells. MDA-MB-231 and MCF-7 cells were seeded into 6-wells and cultured 48 h before transfection. Cells were collected and stained with 10 μL Annexin-V and 10 μL PI for 30 min. Then, the stained cells were analyzed by a flow cytometer (Agilent, Beijing, China).

### Transwell and wound-healing assays

A Transwell chamber with 8 μm pore inserts (BD Falcon, Franklin Lakes, NJ, USA) was used to analyze cell invasion ability. Upper chamber pre-coated with Matrigel matrix (BD Falcon) was added with serum-free medium, and cells (1 × 10^5^/100μL) were cultured into it. The medium contained 10% FBS was added into the low chamber. After 48 h, cells in a low chamber were stained with methyl alcohol (Sigma-Aldrich) and 0.1% crystal violet (Sigma-Aldrich). Then, cells were counted by a microscope (Olympus).

For wound-healing assay, cells (1 × 10^5^ per mL) were fostered in 6-well plates and cultured to 85–90% confluence. The wound was made by 200 μL pipette tips, and then cells were rinsed used water. Subsequently, cells were added 1% FBS medium and cultured for 24 h. A microscope (Olympus) was used to take photos of cells at 0 h and 24 h. Cell migration capacity was evaluated by the wound width.

### Western blot assay

Protein was separated and resolved by sodium dodecyl sulfonate-polyacrylamide gel (SDS-PAGE) and then transferred onto polyvinylidene fluoride (PVDF) membranes (Beyotime). The membranes were blocked with 5% skim milk for 2 h. The primary antibodies as following: anti-matrix metalloprotein 9 (anti-MMP9, ab283575, 1:1000, Abcam, Cambridge, MA, USA), anti-MMP2 (ab181286, 1:1000, Abcam), anti-PGK1 (ab233135, 1:1000, Abcam), anti-β-actin (ab8227, 1:1000, Abcam). The PVDF membranes were incubated in the primary antibodies at 4℃ overnight and then incubated in the secondary antibody for 1 h. The protein bands were visualized by ECL reagent (Beyotime).

### Dual-luciferase reporter assay

The segmental sequences of circHMCU and PGK1 3'UTR containing the wild-type (WT) or mutant (MUT) binding sites of miR-4458 were cloned into psiCHECK2 vector (Geneseed). 293 T cells were seeded into 24-well dishes and individually transfected with psi-CHECK-2-circHMCU wt, psi-CHECK-2-circHMCU mut, psi-CHECK-2-PGK1 3'UTR wt or psi-CHECK-2-PGK1 3'UTR mut together with the miR-4458 mimic or mimic NC using lipofectamine 3000 transfection reagent (Invitrogen). After 48 h transfection, luciferase activities were analyzed by the Dual-Luciferase Reporter Assay Kit (Promega).

### Xenograft mouse model

CircHMCU-shRNA (sh-circHMCU) lentivirus and sh-NC control lentivirus were obtained from Geneseed and used to infect MDA-MB-231 cells as per the manufacturing protocols, respectively. Virus-positive cells were selected by puromycin for 10 days and then were used to establish xenograft mouse model. For xenograft model establishment, a total of 12 female BALB/c nude mice (4–6 week old, 20-22 g) were obtained from Charles River Laboratories (Beijing, China). The mice were randomly divided into two groups (*n* = 6 per group) and injected with MDA-MB-231 cells (3 × 10^6^) transfected with sh-circHMCU or sh-NC into the left flank by subcutaneous injection. Tumor volume was evaluated every week by analyzing the tumor length (L) and width (W). The tumor volume (V) was assessed by the formula V = 1/2(L × W^2^). Four weeks later, the mice were executed, and the tumors were stored in liquid nitrogen or paraffin after weight measurement. The animal studies were approved by the Animal Management Committee of Nanyang Second General Hospital.

### Immunohistochemistry (IHC)

The tumors from mice were paraffin-embedded and cut into 5 μm sections. After antigen retrieval, the sections were blocked in the primary antibody anti-ki-67 (ab279653, 1:1000, Abcam) or anti-PGK1 (ab233135, 1:500, Abcam). The sections were incubated in the second antibody and stained with diaminoaniline (DAB). At last, a fluorescence microscope (Axio Imager A2, Carl Zeiss AG, Germany) was used to take photos.

### Statistical analysis

Data were expressed as the mean ± standard deviation (SD) from at least three independent experiments. The difference between groups was analyzed by the Student's *t*-test or Analysis of Variance (ANOVA) with Tukey's multiple comparisons test. Pearson's correlation coefficients examined the relationship between the two genes. *P < 0.05 was considered a statistically significant difference.

## Results

### CircHMCU was upregulated in BC tissues and cells

**Fig. 1:**
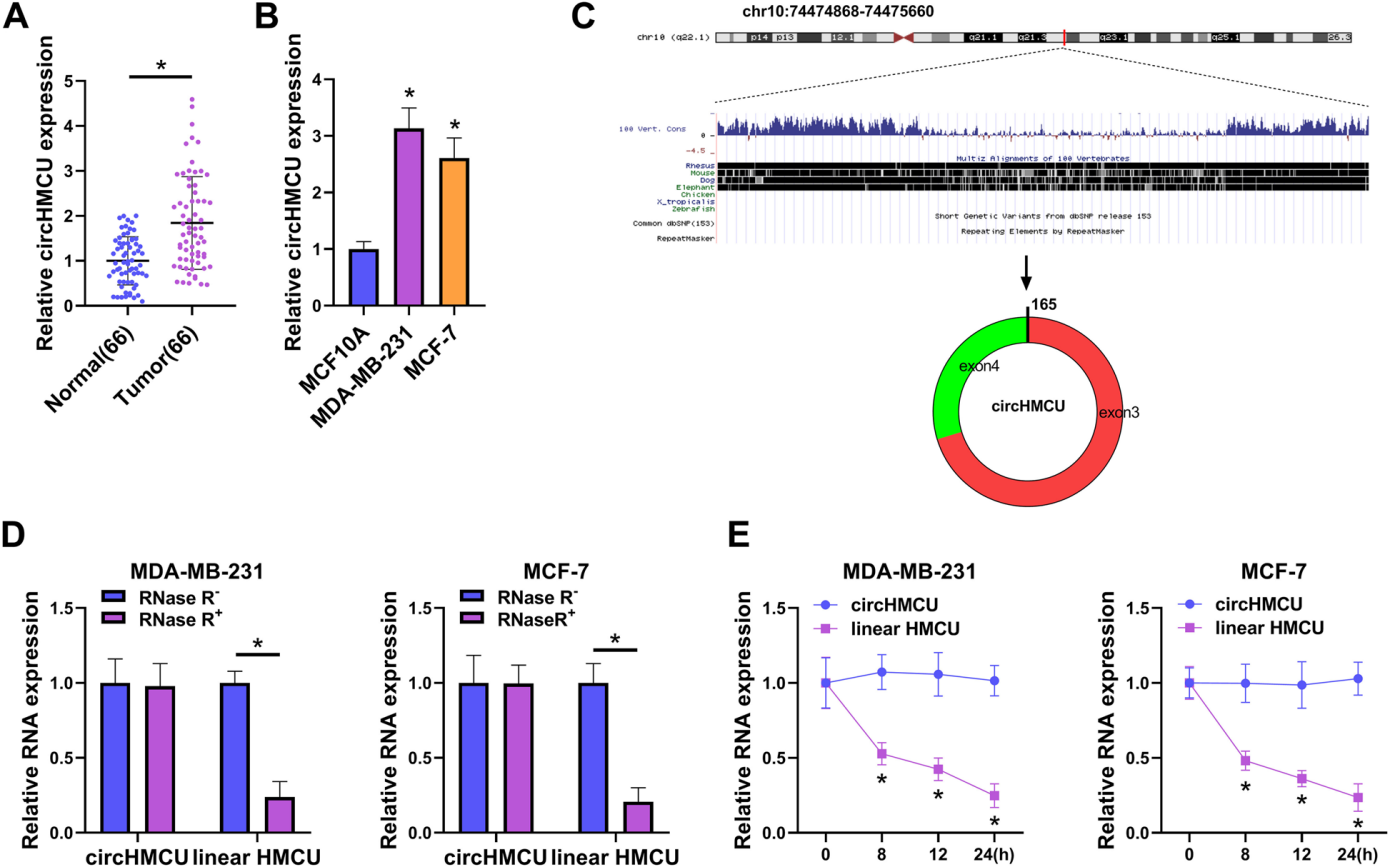
Expression and structure of circHMCU in BC. **A** Expression of circHMCU in Tumor tissues (*n* = 66) and Normal tumor (*n* = 66) was measured by qRT-PCR. **B** The expression of circHMCU in BC cell lines (MDA-MB-231 and MCF-7) and human normal mammary epithelial cell (MCF10A) were determined by qRT-PCR. **C** Schematic diagram showed the back-splicing was constituted with exon 3 and exon 4 in chr10: 74,474,868–74,475,660. **D** After RNase R treatment, the expression of circHMCU and linear HMCU were assessed by qRT-PCR. **E** After Actinomycin D treatment, the levels of circHMCU and linear HMCU were analyzed by qRT-PCR. *P < 0.05

In order to investigate the significance of circHMCU in BC, qRT-PCR experiments were first chose. The results showed that circHMCU was upregulated in BC tumor tissues compared with normal tissues (Fig. 1A). CircHMCU was highly expressed in BC cells compared with MCF10A cells (Fig. 1B). To characterize circular RNA transcripts, we used bioinformatics site (http://www.circbase.org/) and Primer to analyze the structure of circHMCU. We found that circHMCU was generated by back-splicing of exons 3 and 4 of HMCU pre-mRNA (Fig. 1C). The RNase R assay results exhibited that circHMCU was resistant to RNase R, while linear HMCU was digested sharply by RNase R (Fig. 1D). Additionally, actinomycin D treatment showed that circHMCU was more stable than linear HMCU in MDA-MB-231 and MCF-7 cells (Fig. 1E). In short, the findings demonstrated that circHMCU was a head-to-tail loop and aberrantly expressed in BC.

### Suppression of circHMCU expression blocked cell glycolysis, growth, invasion and migration

**Fig. 2:**
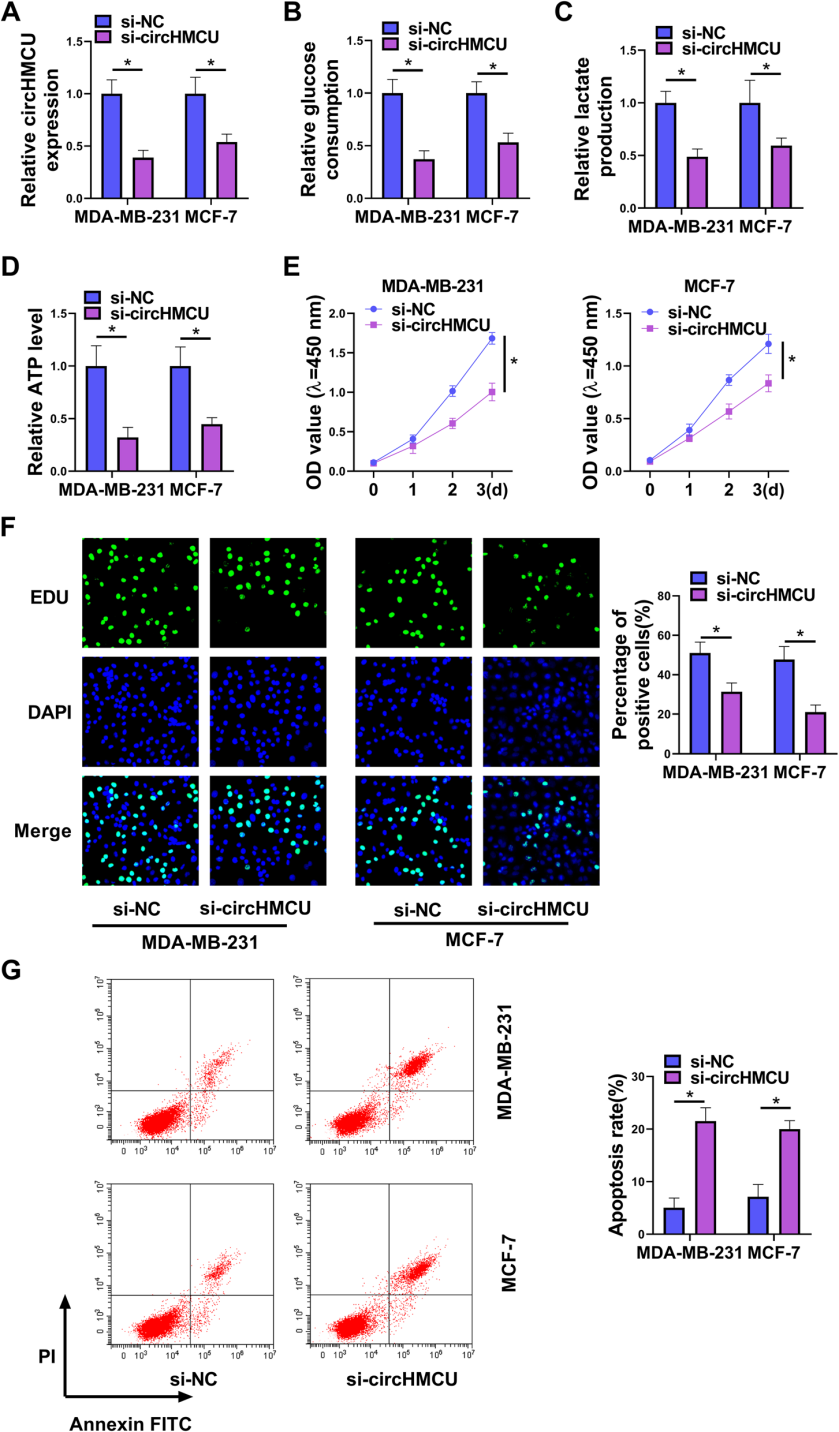
Role of circHMCU in cell glycolysis, growth and apoptosis in BC cells. MDA-MB-231 and MCF-7 cells were transfected with si-NC or si-circHMCU for 48 h. **A** The level of circHMCU was examined by qRT-PCR. **B-D** The levels of glucose consumption, lactate production and ATP were examined with related commercial kits. **E** and **F** Cell proliferation was detected by CCK8 and EdU assays. **G** Cell apoptosis was measured by flow cytometry. *P < 0.05

**Fig. 3:**
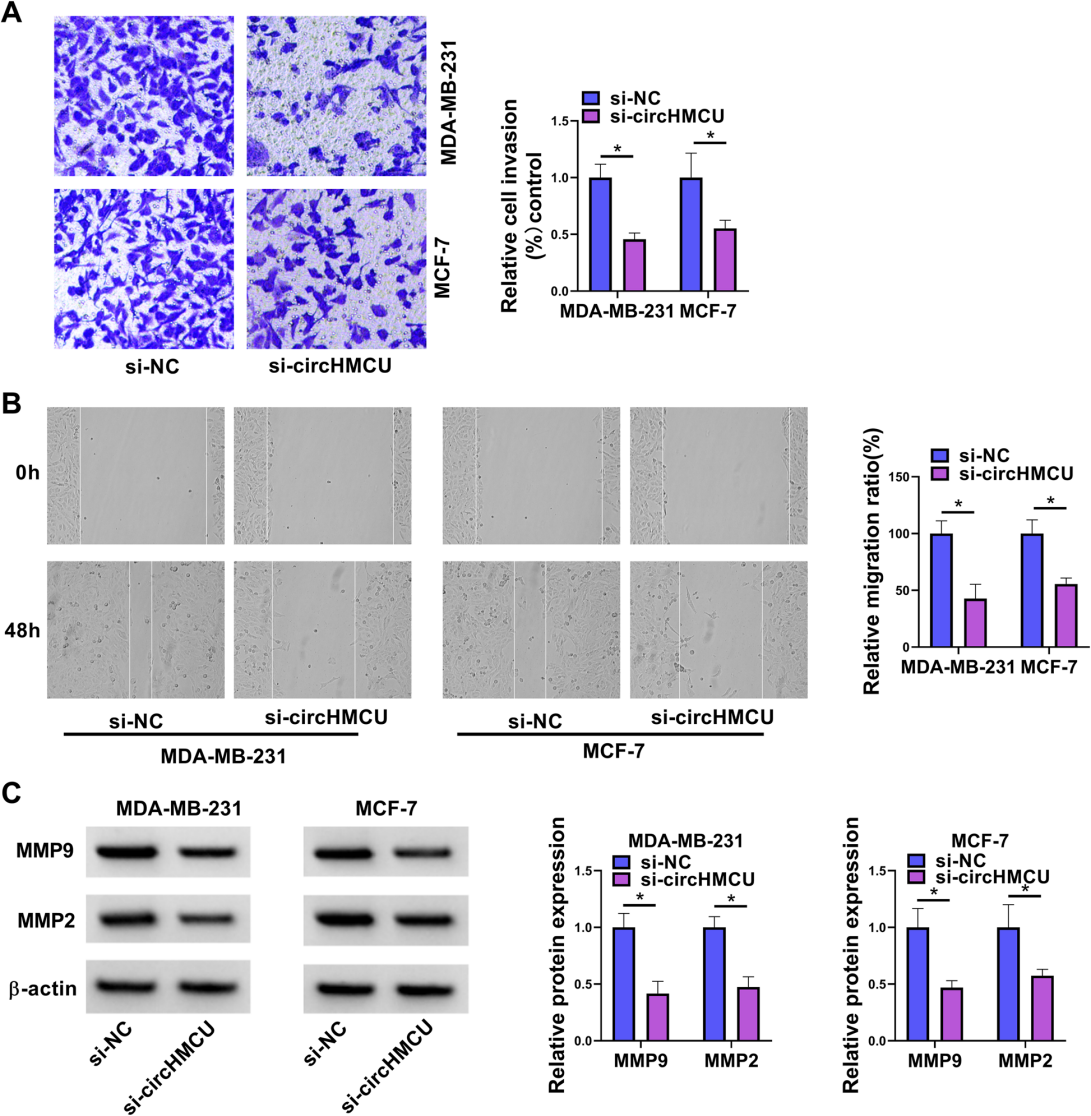
Role of circHMCU in cell invasion and migration in BC cells. MDA-MB-231 and MCF-7 cells were transfected with si-NC or si-circHMCU for 48 h. **A** Cell invasion was tested by transwell assay. **B** Migration was examined by wound-healing assay. **C** The levels of MMP9 and MMP2 proteins were estimated by western blot. *P < 0.05

To investigate the function of circHMCU, we designed and synthesized siRNA to circHMCU (si-circHMCU) and tested the interference efficiency. The results of qRT-PCR presented that circHMCU was downregulated after transfection with si-circHMCU (Fig. 2A). Our results showed that glucose consumption and lactate production were reduced by circHMCU underexpression (Fig. 2B and C). Also, circHMCU depletion inhibited the level of ATP which was measured by ATP assay kit (Fig. 2D). CCK8 assay and EdU assay indicated that knockdown of circHMCU repressed cell proliferation (Fig. 2E and F). In contrast, interfering of circHMCU promoted cell apoptosis (Fig. 2G). The effects of circHMCU on invasion and migration were examined via transwell and wound-healing assays. After transfection of si-circHMCU, the invasion and migration abilities of BC cells were repressed (Fig. 3A and B). In addition, downregulation of circHMCU suppressed the MMP9 and MMP2 levels of MDA-MB-231 and MCF-7 cells (Fig. 3C). Collectively, these results disclosed that circHMCU depletion suppressed cell malignant behaviors in BC cells.

### CircHMCU targeted miR-4458 in BC cells

**Fig. 4:**
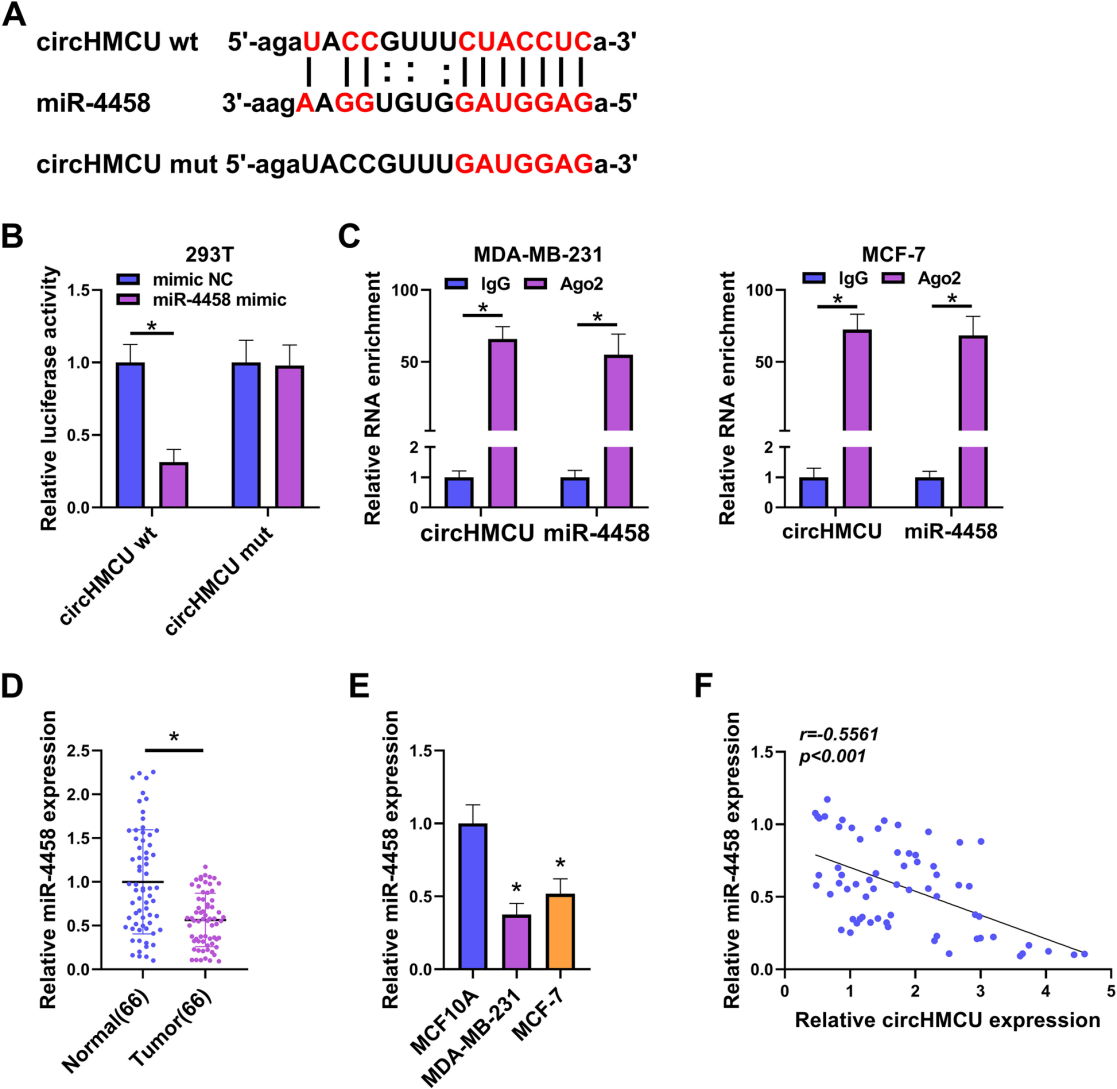
CircHMCU was a sponge of miR-4458. **A** The predicted binding site between circHMCU and miR-4458 was exhibited. **B** and **C** The binding relationship between circHMCU and miR-4458 was verified by dual-luciferase reporter assay and RIP assay. **D** The expression of miR-4458 in normal tissues (*n* = 66) and tumor tissues (*n* = 66) was tested by qRT-PCR. **E** miR-4458 expression was measured in BC cell lines (MDA-MB-231 and MCF-7) and human normal mammary epithelial cell (MCF10A). **F** Pearson analysis was conducted to analyze the correlation between circHMCU and miR-4458 in BC tissues. *P < 0.05

To define the underlying mechanism of circHMCU in regulating BC cell phenotypes, we predicted the potential targeted miRNAs of circHMCU by circbank database (http://www.circbank.cn/). Among these candidates, we selected several miRNAs that are related to BC development and found that miR-4458 expression was the most significantly increased in circHMCU-silenced MCF-7 cells (Supplementary Fig. 1A). We thus focused on miR-4458 in this study. The prediction results showed that circHMCU had a binding site with miR-4458 (Fig. 4A). To further validate the relationship between circHMCU and miR-4458, we executed a dual-luciferase report system and RIP assay. The results displayed that miR-4458 mimic decreased the luciferase activity of circHMCU wt group but not circHMCU mutant group (Fig. 4B). Moreover, circHMCU was significantly pulled down by anti-AGO2 antibody compared with IgG control (Fig. 4C). In addition, miR-4458 was downregulated in the tumor group relative to the normal group (Fig. 4D). The qRT-PCR assay also showed that miR-4458 was downregulated in BC cells (Fig. 4E). Using Spearman’s correlation coefficient, we found that circHMCU expression had a negative relationship with miR-4458 (Fig. 4F). In summary, we illuminated that circHMCU bound to miR-4458 via the pairing sites.

### The effects of circHMCU depletion on BC cell phenotypes were abrogated by inhibition of miR-4458

**Fig. 5:**
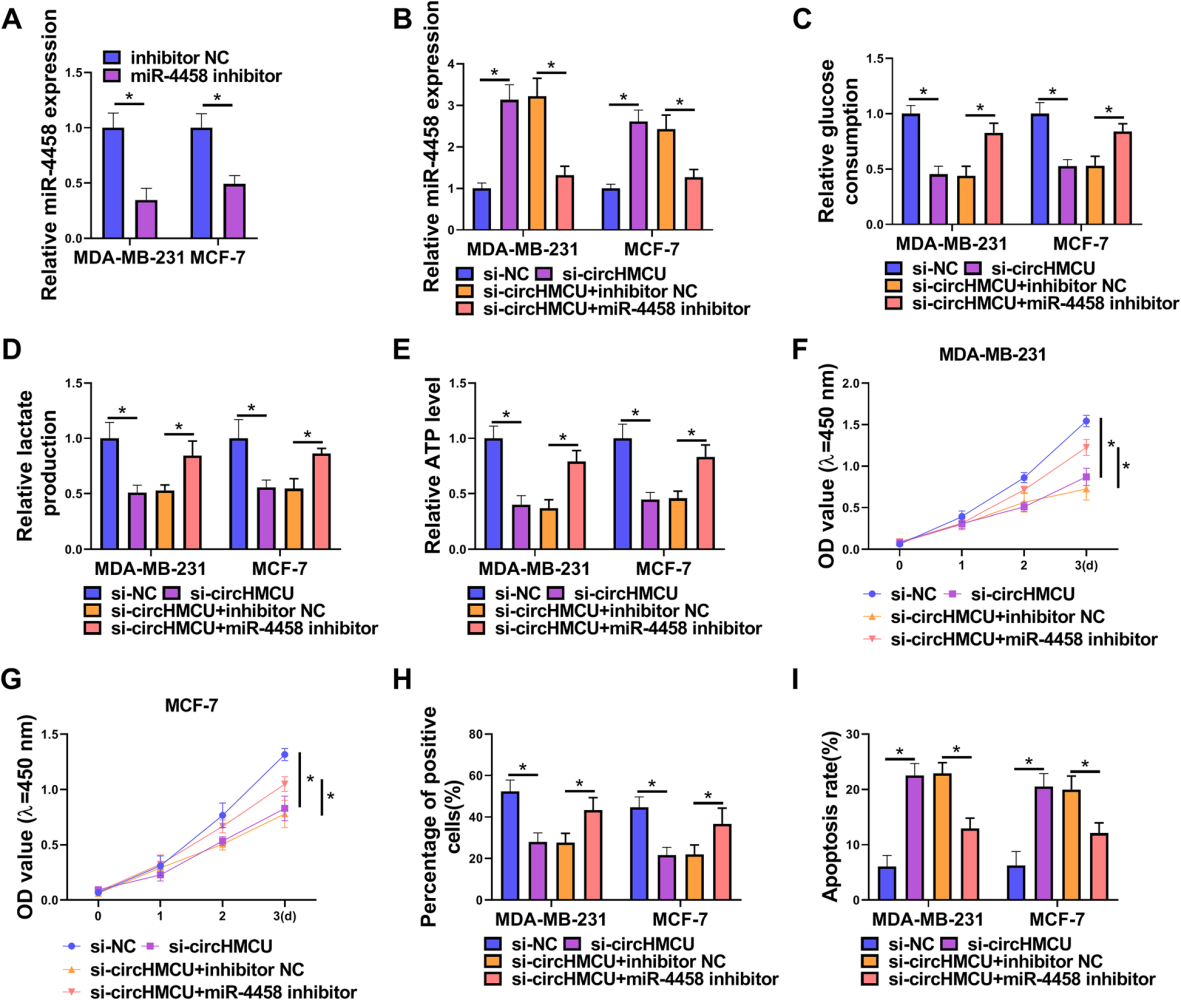
Knockdown of miR-4458 reversed the effects of circHMCU on BC cell glycolysis, growth and apoptosis. MDA-MB-231 and MCF-7 cells were transfected with si-NC, si-circHMCU, si-circHMCU + inhibitor NC, or si-circHMCU + miR-4458 inhibitor. **A** and **B** qRT-PCR measured miR-4458 expression. **C-E** Commerial kits were used to measure glucose consumption, lactate production and ATP level. **F** and **G** Cell proliferation was detected by CCK8 and EdU assay. **H** Cell apoptosis was measured by flow cytometry. **I** Cell invasion was tested by transwell assay. *P < 0.05

**Fig. 6:**
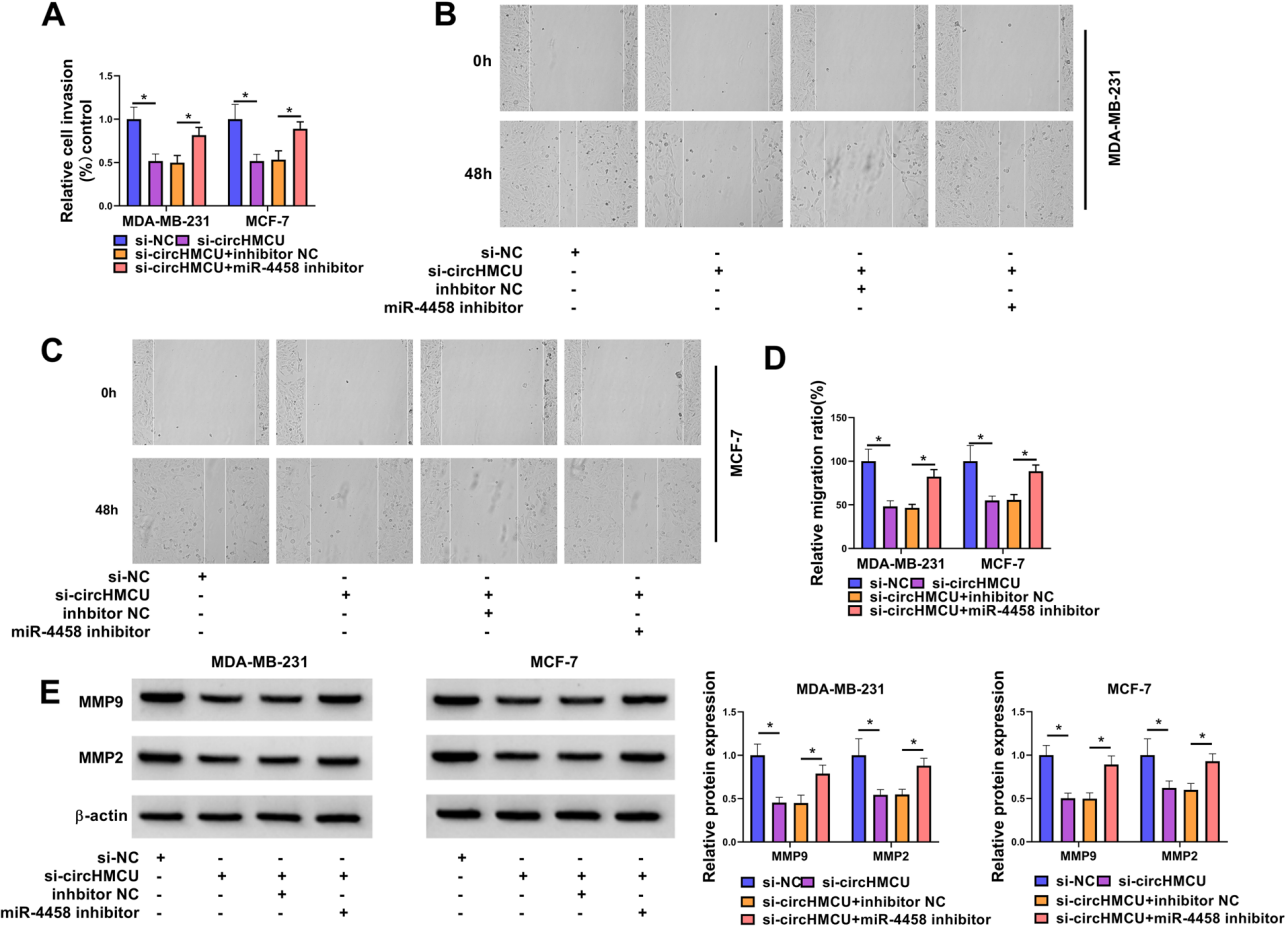
Knockdown of miR-4458 reversed the effect of circHMCU on BC cell invasion and migration. MDA-MB-231 and MCF-7 cells were transfected with si-NC, si-circHMCU, si-circHMCU + inhibitor NC, or si-circHMCU + miR-4458 inhibitor. **A** Cell invasion was tested by transwell assay. **B-D** Migration was examined by wound-healing assay. **E** The levels of MMP9 and MMP2 proteins were estimated by western blot. *P < 0.05

To explore whether circHMCU could affect the biological behaviors of BC cells via miR-4458, we performed miR-4458 silencing experiments in si-circHMCU-transfected cells. After BC cells were transfected with miR-4458 inhibitor, the levels of miR-4458 were downregulated (Fig. 5A). The qRT-PCR experiments demonstrated that the expression of miR-4458 was elevated after knockdown of circHMCU, while the effect was restored by miR-4458 inhibitor (Fig. 5B). In glycolysis metabolism assays, circHMCU depletion curbed glucose consumption, lactate production and ATP level, which were recuperated by miR-4458 inhibitor (Fig. 5C-E). The results of CCK8 assay and EdU assay showed that down-expression of miR-4458 weakened the inhibitory effect of circHMCU depletion on cell growth capability (Fig. 5F-H). Suppression of circHMCU facilitated cells apoptosis, which was partly abated after co-transfection with miR-4458 inhibitor into BC cells (Fig. 5I). In addition, we found that miR-4458 inhibitor reversed the effect of circHMCU knockdown on cell invasion and migration (Fig. 6A-D). Moreover, Western blot assay showed that miR-4458 inhibitor restored the inhibitory effect of circHMCU knockdown on MMP9 and MMP2 levels (Fig. 6E). Herein, we concluded that circHMCU affected BC tumor progression via regulation of miR-4458.

### CircHMCU regulated PGK1 through miR-4458

**Fig. 7:**
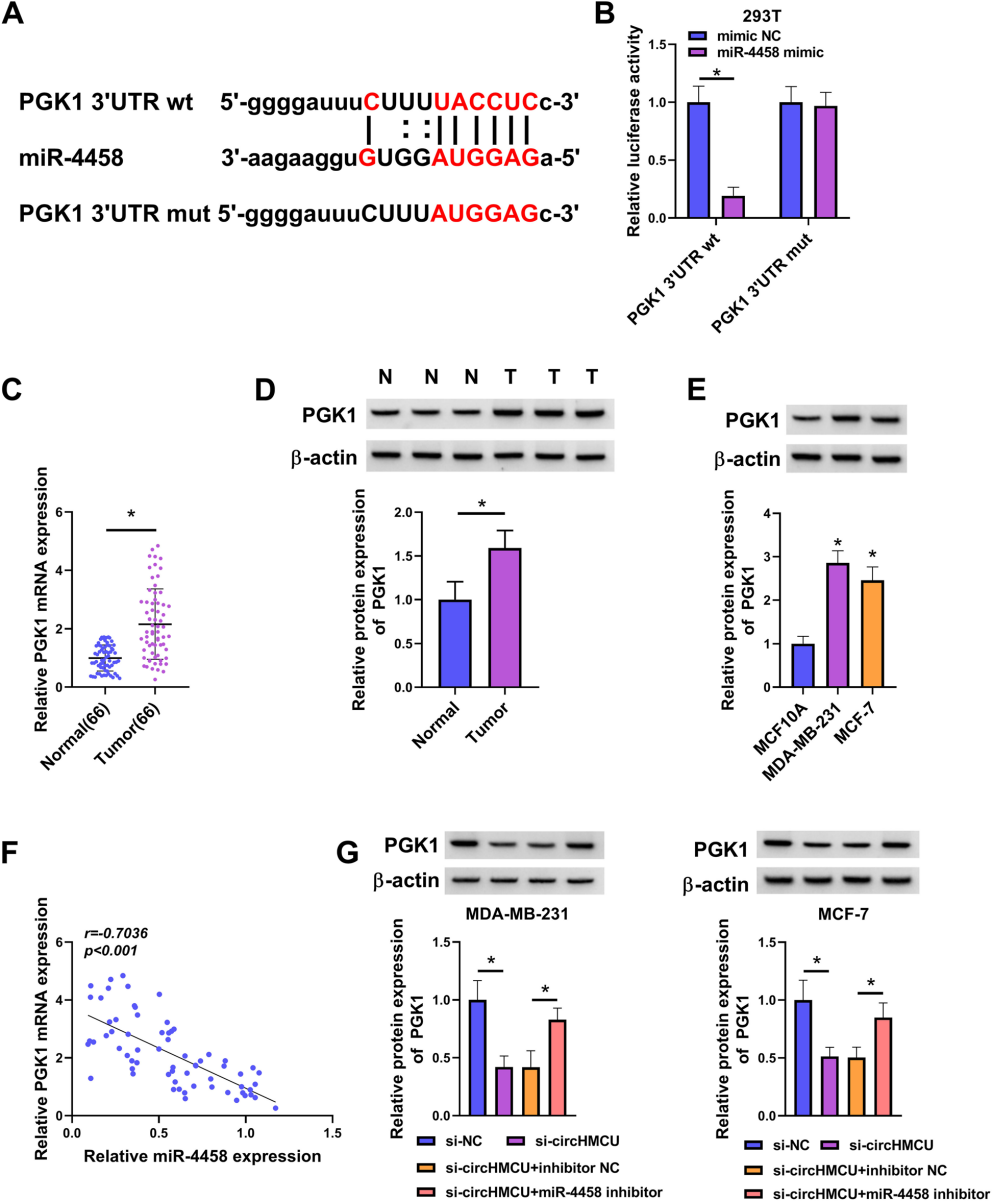
MiR-4458 directly target PGK1. **A** The predicted binding site between miR-4458 and PGK1was exhibited. **B** The binding relationship between miR-4458 and PGK1 was verified by dual-luciferase reporter assay. **C** The expression of PGK1 in normal tissues (*n* = 66) and tumor tissues (*n* = 66) was tested by qRT-PCR. **D** The expression of PGK1 protein in in normal tissues and tumor tissues was tested by western blot. **E** PGK1 protein expression was measured in BC cell lines (MDA-MB-231 and MCF-7) and human normal mammary epithelial cell (MCF10A). **F** Pearson analysis was conducted to analyze the correlation between miR-4458 and PGK1 in BC tissues. **G** The expression of PGK1 protein was exhibited by western blot.*P < 0.05

To define the downstream effectors of miR-4458, we used starbase software to search its mRNA targets. Among these predicted targets, we selected six genes that exert crucial functions in BC development and observed that PGK1 mRNA expression was the most significantly down-regulated in MCF-7 cells after transfection by miR-4458 mimic (Supplementary Fig. 1B). As illustrated in Fig. 7A, miR-4458 contained a complementary region for PGK1 3’UTR. The dual-luciferase report activity showed that miR-4458 mimic repressed the luciferase activity of PGK1 3’UTR wt but not PGK1 3’UTR mutant, suggesting that miR-4458 could interact with PGK1 (Fig. 7B). The qRT-PCR tests showed that PGK1 was highly expressed in tumor tissues (Fig. 7C). Consistently, the overexpression of PGK1 protein was confirmed in tumor tissues and cells by western blot (Fig. 7D and E). Also, we discovered that there was a negative correction between miR-445 and PGK1 expression in BC tissues (Fig. 7F). Furthermore, we confirmed that knockdown of miR-4458 rescued the downregulation of PGK1 in si-circHMCU-transfected BC cells (Fig. 7G). Collectively, the data showed that circHMCU enhanced PGK1 expression via regulating miR-4458.

### The effects of miR-4458 overexpression on BC cells were reversed by PGK1 re-expression

**Fig. 8:**
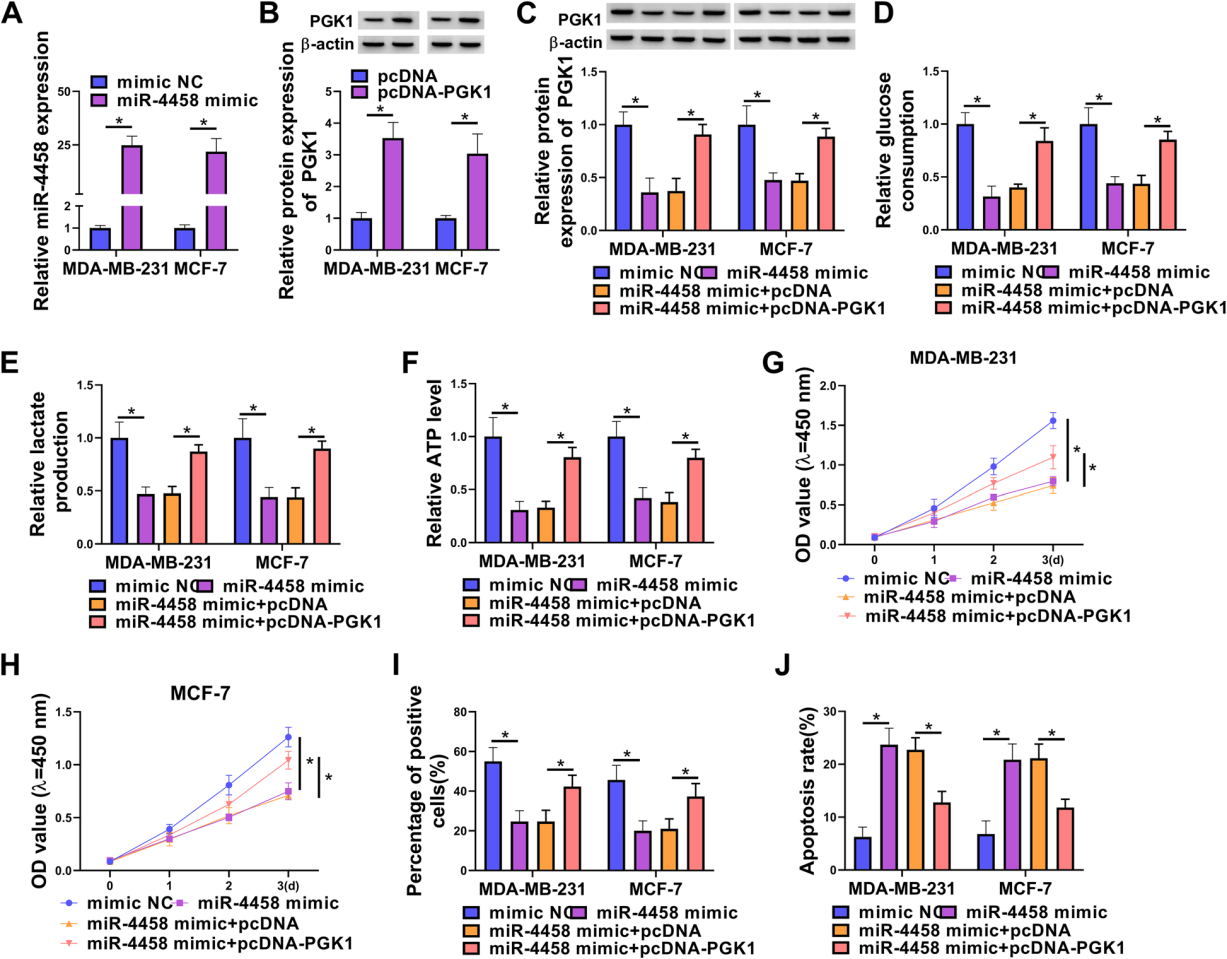
PGK1 overexpression reversed the effects of miR-138-5p mimic on BC cell glycolysis, growth and apoptosis. **A** qRT-PCR measured miR-4458 expression. **B** Western blot measured the expression of PGK1. **C-J** MDA-MB-231 and MCF-7 cells were transfected with mimic NC, miR-4458 mimic, miR-4458 mimic + pcDNA, or miR-4458 mimic + pcDNA-PGK1. **C** Western blot measured the expression of PGK1. **D-F** Commerial kits measured the levels of glucose consumption, lactate production and ATP. **G-I** Cell proliferation was detected by CCK8 and EdU assay. **J** Cell apoptosis was measured by flow cytometry. *P < 0.05

**Fig. 9:**
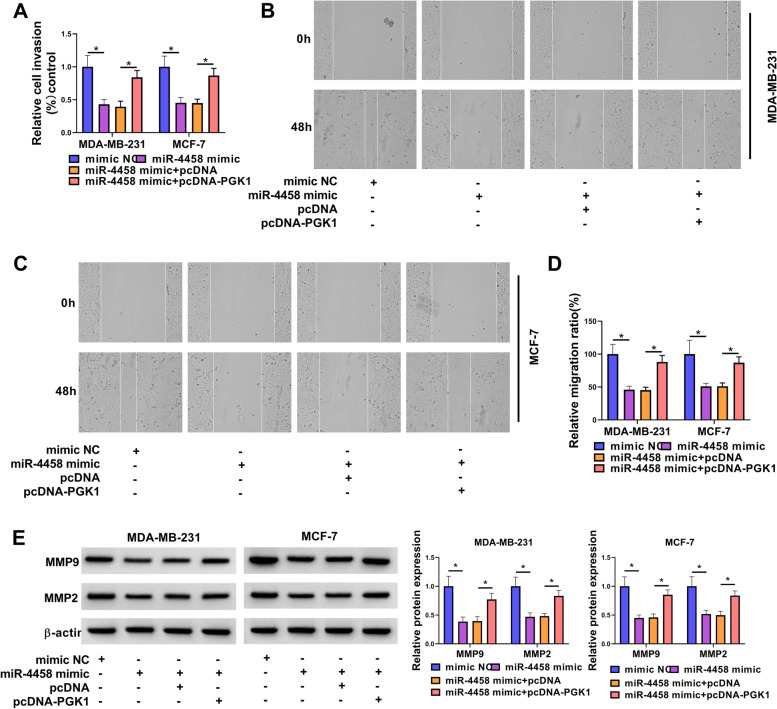
PGK1 overexpression reversed the effects of miR-138-5p mimic on BC cell invasion and migration. MDA-MB-231 and MCF-7 cells were transfected with mimic NC, miR-4458 mimic, miR-4458 mimic + pcDNA, or miR-4458 mimic + pcDNA-PGK1. **A** Cell invasion was tested by transwell assay. **B-D** Migration was examined by wound-healing assay. **E** The levels of MMP9 and MMP2 proteins were estimated by western blot. *P < 0.05

To further verify whether miR-4458 exerted its inhibitory effect on BC by directly targeting PGK1, we performed a rescue experiment to examine the functional interaction of miR-4458 and PGK1. The expression of miR-4458 was upregulated after transfection by miR-4458 mimic (Fig. 8A). The western blot assay showed that PGK1 protein was upregulated by transfection with pcDNA-PGK1 (Fig. 8B). Overexpression of PGK1 rescued miR-4458 mimic-caused decrease on PGK1 expression (Fig. 8C). We also found that miR-4458 mimic inhibited glucose consumption, lactate production and ATP level of BC cells, whereas overexpression of PGK1 restored these repressions (Fig. 8D-F). The results displayed that cell proliferation was weakened by miR-4458 mimic, but this effect was impaired by transfection with pcDNA-PGK1 (Fig. 8G-I). Conversely, flow cytometry assays revealed that cell apoptosis ratio was enhanced by miR-4458 mimic, which was abolished by ectopic expression of PGK1 (Fig. 8J). Transwell and wound-healing assays showed that cell invasion and migration abilities were also repressed by miR-4458 mimic, which were regained by overexpression of PGK1 (Fig. 9A-D). Moreover, overexpression of PGK1 also abrogated miR-4458 mimic-induced decrease on MMP9 and MMP2 expression (Fig. 9E). Collectively, the results showed that miR-4458 played a tumor-inhibiting function in BC cells by binding and inhibiting PGK1.

### CircHMCU depletion weakened tumor growth in vivo

**Fig. 10:**
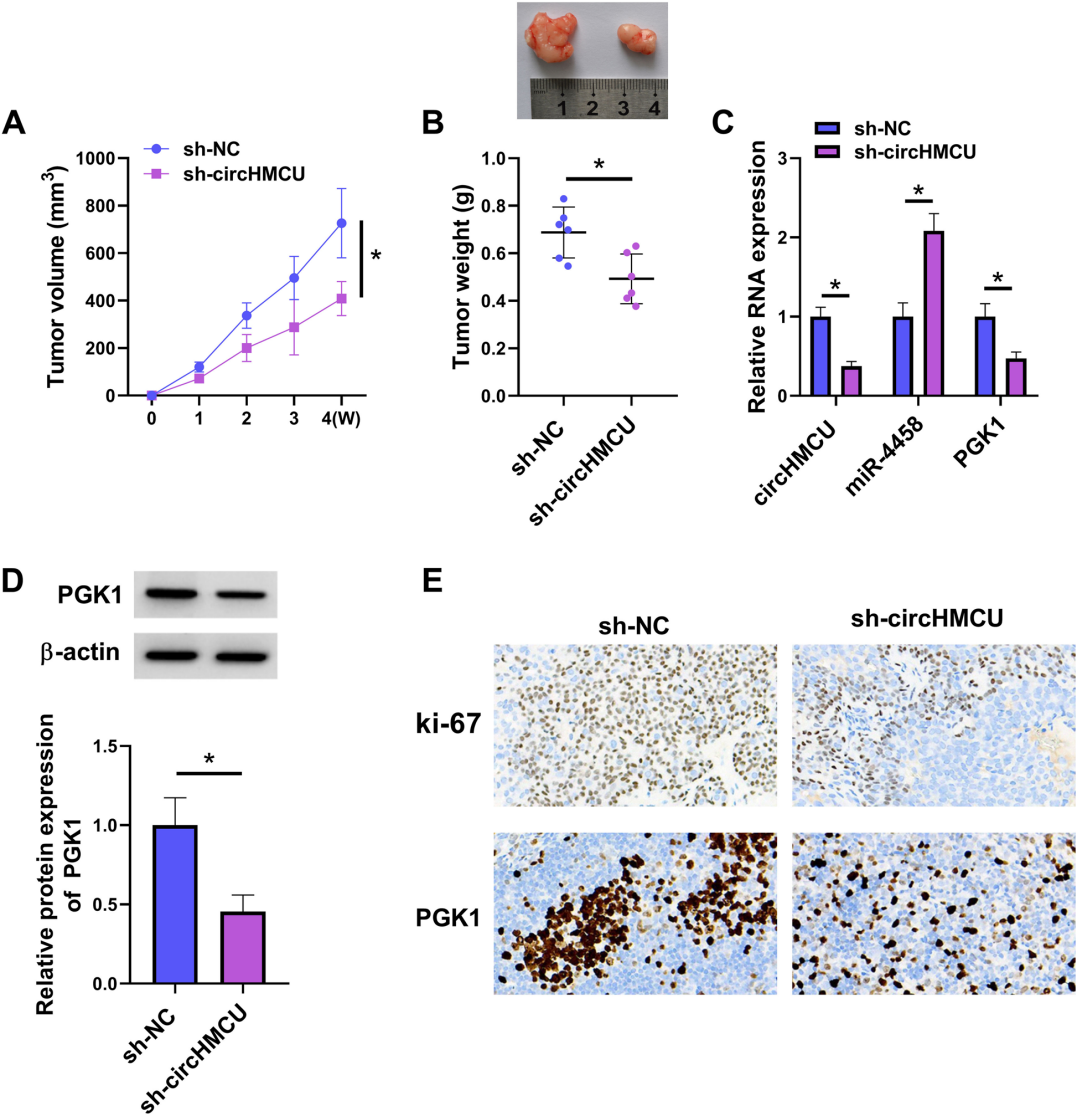
Silencing of circHMCU receded tumour growth in vivo. MDA-MB-231 cells were transfected with sh-NC or sh-circHMCU. **A** Tumor volume was measured every week. **B** Tumor weight was examined after mice were sacrificed. **C** The levels of circHMCU, miR-4458 and PGK1 were measured by qRT-PCR. **D** The protein level of PGK1 was assessed by western blot. **E** The levels of ki-67 and PGK1 were detected by IHC. *P < 0.05

To determine the effect of circHMCU on tumor growth, MDA-MB-231 cells stably transfected with sh-NC or sh-circHMCU were injected into female nude mice. The tumor volume and weight were reduced in sh-circHMCU group compared with sh-NC group (Fig. 10A and B). The expression levels of circHMCU and PGK1 were downregulated in the sh-circHMCU group, while miR-4458 expression was increased (Fig. 10C and D). Moreover, the IHC assay showed that knockdown of circHMCU decreased ki-67 and PGK1 levels (Fig. 10E). In summary, the results suggested the tumor-inhibitory function of circHMCU depletion in vivo.

## Discussion

Our results showed that circHMCU and PGK1 were upregulated, whereas miR-4458 was downregulated in BC tumors and cells. We also confirmed that circHMCU had a binding site with miR-4458 by dual-luciferase activity report and RIP assays. The targeting correction of miR-4458 and PGK1 was also confirmed in this study. Furthermore, we first demonstrated that high expression of circHMCU enhanced BC cell malignant phenotypes in vitro and tumor growth in vivo by regulating miR-4458/PGK1 axis.

CircRNAs play tumor-inhibiting effects or tumor-promoting effects in BC [25, 26]. For instance, circSKA3 combined integrin β1 to cause invadopodium formation boosting tumor progress in BC [27]. The upregulation of circ-Dnmt1 could activate oncogenic proteins to accelerate the proliferation of BC cells by enhancing cellular autophagy [28]. In the current study, using a series of cell functional analyses, we found that circHMCU (has_circ_0000247), a 792 bp exonic circRNA formed by back-splicing of exons of its parental gene HMCU, was highly expressed in BC. In line with a previous report [13], our results also showed that circHMCU were overexpressed in BC tumors, and depletion of circHMCU hindered cancer cell malignant phenotypes in vitro and tumor growth in vivo.

The miRNA sponge mechanism, as the one regulatory pathway of circRNAs, has been confirmed in various tumor cells, such as colorectal cancer [29], lung cancer [30], hepatocellular carcinoma [31]. We used bioinformatics, dual-luciferase activity report and RIP assay testified that circHMCU could bind to miR-4458. Some researchers have reported the functions of miR-4458 in BC. For instance, miR-4458 hampered the growth and metastasis of BC by targeting CPSF4 [15]. MiR-4458 hindered cell migration and proliferation by reducing SOCS1 in BC cells [16]. Our results also suggested that miR-4458 acted as a tumor inhibitor in BC, and its inhibition significantly reversed the effects of circHMCU depletion on cell glycolysis, proliferation, apoptosis and motility. Therefore, circHMCU may augment the overexpression of downstream genes by regulating miR-4458, thereby augmenting BC progression.

PGK1, a member of PGK family, is ubiquitously expressed in almost all cells [32]. PGK1 is a key enzyme gene of glycolysis, where it stimulates the translation of 1,3-diphosphoglyceride into 3-phosphoglycerate and produces a molecule of ATP [33, 34]. In addition, PGK1 enhances glucose uptake and lactate production in tumor cells [35]. At the same time, the high expression of PGK1 is found in multiple cancers and is corrected with the poor prognosis of cancer [36, 37]. Moreover, some findings demonstrated that PGK1 was upregulated in various types of BC [17, 18]. MiRNA-16–1-3p hindered BC cells migratory and invasive abilities by inhibiting PGK1-regulated glycolysis [38]. PGK1 has established a potential tumor promoting role by boosting the HIF-1α expression to stimulate tumor metastasis in BC [19]. Consistent with these findings, our results validated that the expression of PGK1 was elevated in BC tissues and cells. Furthermore, we uncovered that miR-4458 acted as a tumor inhibitor in BC through PGK1. More importantly, we showed, for the first time, that circHMCU involved the post-transcriptional regulation of PGK1 via miR-4458.Taken together, circHMCU knockdown inhibited cell proliferation, migration, invasion and glycolysis while induced cell apoptosis in BC cells by miR-4458/PGK1 axis. The study suggested that circHMCU might be an underlying prognostic biomarker for BC.

## Supplementary Information

**Additional file 1: Supplementary Figure 1.** Expression of circHMCU in clinical samples and selection of miR-4458 and PGK1. (A) Expression of eight miRNAs by qRT-PCR in MCF-7 cells transfected with si-circHMCU or si-NC. (B) Expression of six mRNAs by qRT-PCR in MCF-7 cells transfected with mimic NC or miR-4458 mimic. *P < 0.05.

## Data Availability

The data that support the findings of this study are available from the corresponding author upon reasonable request.

## Abbreviations

BC: Breast cancer
circRNAs: Circular RNAs
PGK1: Phosphoglycerate kinase 1
qRT-PCR: Quantitative real-time polymerase chain reaction
CCK8: Cell Counting Kit-8
IHC: Immunohistochemistry
