# Supplementary figures and images for: Circular RNA circHMCU promotes breast tumorigenesis through miR-4458/PGK1 regulatory cascade
Source: Hereditas. 2023 Mar 23;160:12. doi: 10.1186/s41065-023-00275-y (PMC10035165; doi:10.1186/s41065-023-00275-y)

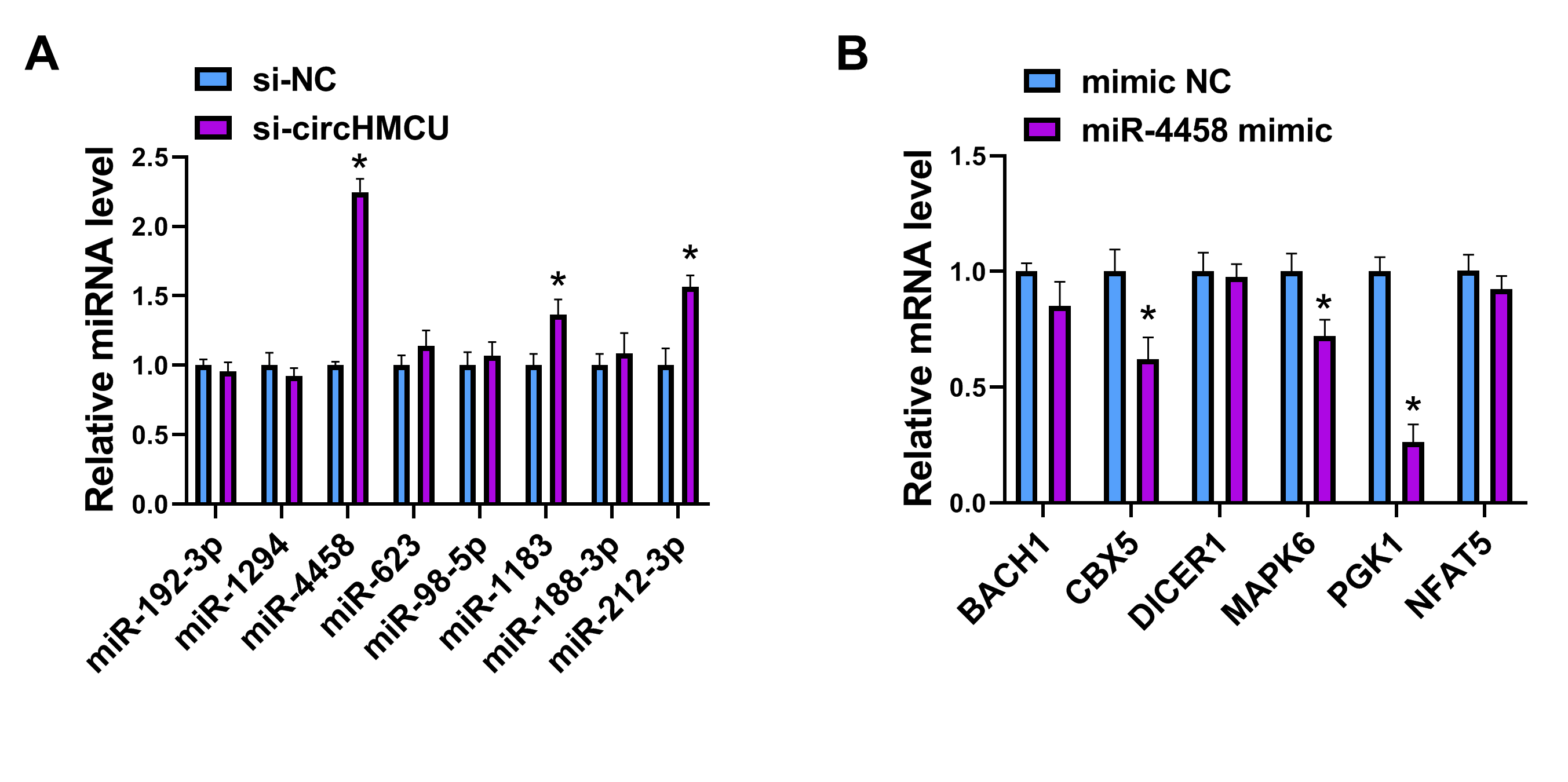

Supplement: Supplementary file 1 — Additional file 1: Supplementary Figure 1. Expression of circHMCU in clinical samples and selection of miR-4458 and PGK1. (A) Expression of eight miRNAs by qRT-PCR in MCF-7 cells transfected with si-circHMCU or si-NC. (B) Expression of six mRNAs by qRT-PCR in MCF-7 cells transfected with mimic NC or miR-4458 mimic. *P < 0.05. [file 41065_2023_275_MOESM1_ESM.tif]
